# Effectiveness of Dry Needling Versus Percutaneous Electrolysis in Achilles Tendinopathy: A Randomized Clinical Trial

**DOI:** 10.7759/cureus.104384

**Published:** 2026-02-27

**Authors:** Sara Delgado Álvarez, Roberto Méndez Sánchez, Luis Polo Ferrero, Isaac Rodríguez Fragua, Catalina Loaiciga Espeleta, Roberto González Raja, Zacarías Sánchez Milá, Jorge Velázquez Saornil

**Affiliations:** 1 Department of Physiotherapy, Universidad Católica de Ávila, Ávila, ESP; 2 Department of Physiotherapy, Universidad de Salamanca, Salamanca, ESP; 3 Department of Physiotherapy, Universidad Pontificia de Salamanca, Salamanca, ESP; 4 NEUMUSK Group Research, Department of Physiotherapy, Faculty of Health Sciences, Avila Catholic University, Castile and León, ESP

**Keywords:** achilles tendinopathy, algometry, dry needling, functionality, pain, physiotherapy, quality of life, range of motion, ultrasound-guided percutaneous electrolysis

## Abstract

Introduction: Achilles tendinopathy (AT) is characterized by pain, inflammation, and functional limitations. It is also the most common cause of pain located at the back of the calcaneus. The Achilles tendon is among the most vulnerable tendons in the lower limb, and its pathology is one of the most common overuse injuries. Moreover, it is not an injury exclusive to athletes, as 65% of diagnosed Achilles tendinopathies are not related to sport.

Methods: Randomized, single-blind clinical trial with 60 patients, 30 in each group. All study participants were previously diagnosed with insertional AT, mid-portion tendinopathy, or both and were referred by an orthopedic surgeon. The short- and medium-term results of treatment with dry needling (DN) application to the gastrocnemius muscle trigger points (MTrPs) versus ultrasound-guided percutaneous electrolysis (PE) application to the Achilles tendon were observed in terms of pain intensity, pressure pain threshold, ankle dorsiflexion range of motion under load, quality of life, and ankle and foot function in patients with previously diagnosed AT.

Results: The visual analogue scale revealed that the time effect was statistically significant. Similarly, AT algometry revealed that the time effect was statistically significant, indicating that the pressure pain threshold assessed by algometry changed significantly over the course of the study. Moreover, quality of life and functionality showed statistically significant improvements from the third week of the study, whereas range of motion remained unchanged.

Conclusion: Ultrasound-guided PE applied to the Achilles tendon is more effective than DN applied to the MTrPs of the gastrocnemius muscle in reducing pain intensity and improving quality of life and ankle and foot function in the short and medium term in patients with AT.

## Introduction

Achilles tendinopathy (AT) is characterized by localized pain, inflammation, and impaired function [1]. It can be classified anatomically into insertional tendinopathy, involving the bony insertion into the calcaneus, or noninsertional tendinopathy, also called mid-portion tendinopathy. Janssen et al. [2] reported that 43% of elite athletes had current or previous symptoms of AT, with middle-distance runners being the most affected, with a prevalence of 83%, influencing athletic performance. This pathology is a common cause of disability that affects both the active population, in which 52% of runners have suffered this injury during their sporting career, and the inactive population, in which 65% of diagnosed AT cases are not related to sports. Invasive physiotherapy techniques such as dry needling (DN) and percutaneous electrolysis (PE) are receiving increasing evidence [3-5]; however, no studies have investigated which technique is more effective for this type of injury.

Conservative treatment based on a combination of eccentric exercise and stretching has been shown to be the treatment of choice early in the injury for pain management in tendinopathies [6]. The presence of muscle trigger points (MTrPs) in the medial head of the gastrocnemius muscle tends to lead to pain in the ankle and the medial and posterior aspects of the Achilles tendon [3-5], and DN provides strong evidence for the management of this injury [7]. In addition, DN associated with eccentric exercise and stretching has been found to be more effective than either intervention alone in the treatment of tendinopathies [8,9].

The etiology of tendinopathies is not well understood but is thought to be multifactorial. It is considered a failed healing response with characteristic changes in tenocytes, disorganization of collagen fibers, increased extracellular matrix, and neovascularization [10]. PE, accompanied by eccentric exercise and stretching, owing to mechanotransduction, can produce changes in the extracellular matrix, fibroblasts, and collagen fiber junction bridges [11] and is also a useful technique for the management of tendinopathies [12,13].

One of the main reasons for the high incidence rate of this injury, which is between 27% and 44%, is that only symptoms are considered. Therefore, this research aims to determine which of these methods, PE or DN, is more effective for the management of pain, function, range of motion, and quality of life in patients with AT in the short and medium term.

## Materials and methods

A randomized, comparative, single-blind (evaluator blinded) clinical trial is being conducted in patients with AT, diagnosed by a traumatologist. The variables studied were Visual Analogue Scale (VAS) score [14], pain threshold to pressure (algometry), ankle dorsiflexion range of motion (goniometry), quality of life (Victorian Institute of Sport Assessment-Achilles (VISA-A)) [15], and ankle and foot function (Functional Scale of the Lower Limb and the Measure of the Ankle and Foot Capacity (FAAM)) [16]. These variables were measured during five evaluations throughout the study (preintervention, one week, two weeks, three weeks, and one month).

The inclusion criteria were signed informed consent to participate in the study and to undergo invasive physiotherapy techniques; presence of symptoms (swelling or pain) ≥ 4 weeks; positive Achilles tendon palpation test; male and female individuals over 18 years of age; presence of MTrPs in the gastrocnemius muscle, meeting at least three of the following diagnostic criteria described by Travell and Simons [17]; the presence of a palpable tense band; local pain on pressure at the nodule of the tense band; recognition by the patient as their usual pain after mechanical stimulation of the tender nodule; and limited range of motion (ROM).

The exclusion criteria were any related acute or chronic musculoskeletal disease, which may affect the results of the study; the presence of neuropathic pain such as lumbar radiculopathy, which may affect the results of the study; failure to receive all 4 study treatment sessions and assessments; previous Achilles tendon surgery, ankle arthrodesis or hindfoot fracture; heterometrics; pregnancy; cardiovascular pathology; neurological pathology; local infection or cancer and belonephobia.

Once the study participants have been recruited and after assessment, those who meet the selection criteria are randomly assigned by an independent researcher to the study via the Epidat 3.1 software (Dirección Xeral de Saúde Pública, Xunta de Galicia, Santiago de Compostela, Spain) to one of two study groups: the DN group or the PE group.

The research is registered on ClinicalTrials.gov under the number NCT06080334. Furthermore, compliance with the Declaration of Helsinki, the Biomedical Law, and the Organic Law on Data Protection is noted.

The sample size calculation accounts for the main variable, pain intensity. The sample size is calculated using the GRANMO Version 7.11 tool (Institut Municipal d’Investigació Mèdica (IMIM), Barcelona, Spain), through analysis of variance of repeated measures of intra- and intergroup interactions with an α risk of 0.05, a β risk of 0.10, a bilateral contrast of two groups (DN and PE), and two measurement times (before and during the week of the intervention). In addition, a study was conducted with a correlation of 0.75, a non-sphericity correction of Ɛ = 1, and a common standard deviation of 3 points. Considering a 10% loss of participants in the follow-up, at least 54 participants are needed, 27 from each group.

DN group intervention

Identification of Latent MTrPs

Without being aware of the treatment assignment, the same physiotherapist identified latent MTrPs in the gastrocnemius muscle when measurements were taken before and after the intervention. The researcher who executed both interventions confirmed the presence of latent MTrPs. The most hyperalgesic latent MTrPs in the gastrocnemius were selected and permanently marked with a cross [17]. A latent MTrP is defined as a hyperirritable nodule within a taut band that is activated or produces pain upon palpation by digital compression and results in limitation of joint range upon stretching [17].

Invasive Technique: DN Group (N = 40)

**Figure 1:**
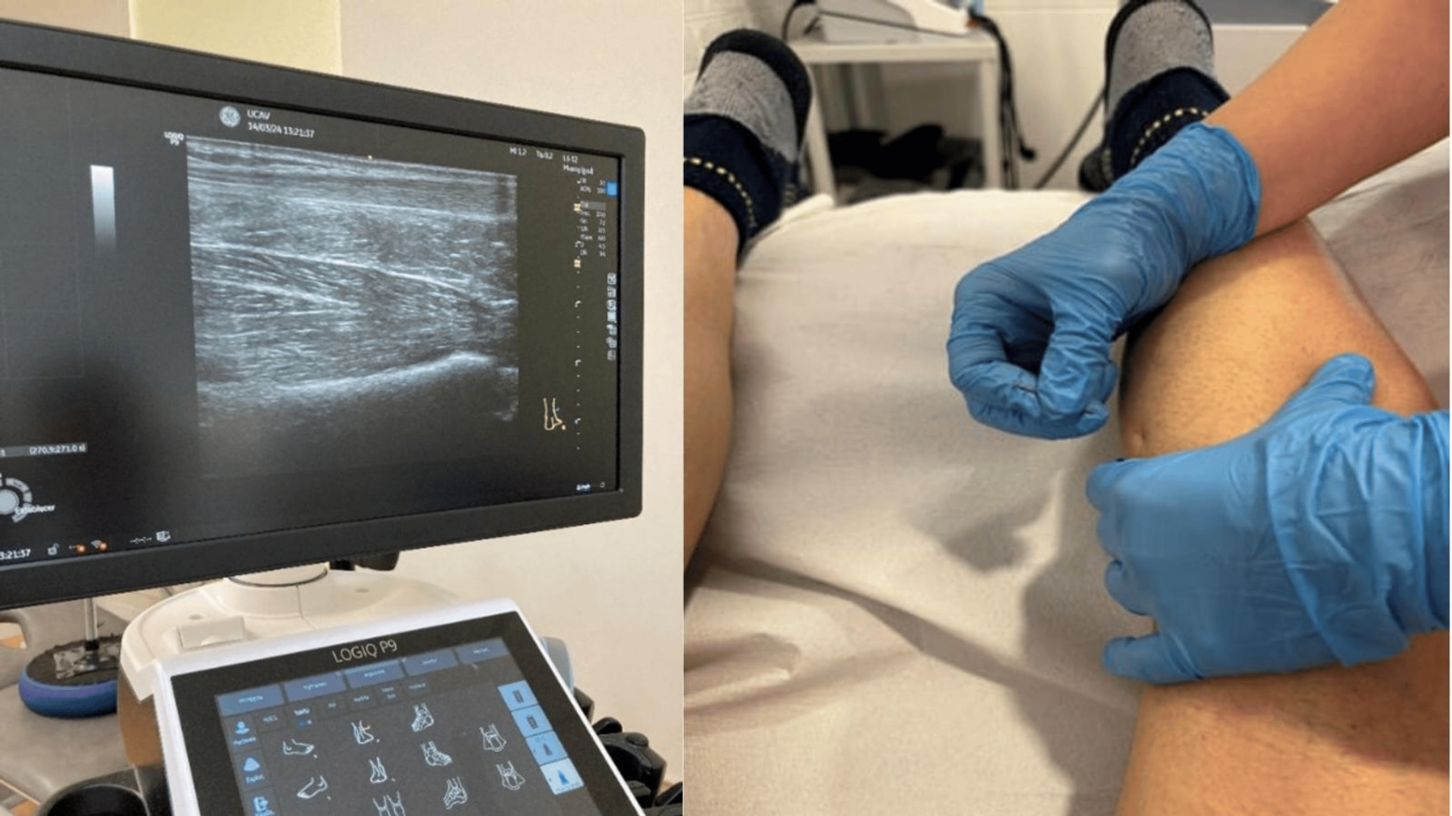
Invasive DN technique. DN: dry needling.

To introduce a 0.25 × 60 mm stainless steel needle, the patient was first positioned in a prone position by the physiotherapist in a sterile environment (Agupunt, Madrid, Spain). The most hyperalgesic latent MTrPs of the gastrocnemius, defined as hyperirritable nodules within a taut band that are activated or produce pain upon palpation by digital compression and result in limitation of joint range upon stretching, were selected and marked with a cross using a permanent marker. The next step was to perform Hong’s entry and exit technique over the MTrP of the gastrocnemius using an invasive method. This intervention is shown in Figure 1.

Ultrasound-Guided Percutaneous Electrolysis Technique: PE Group (N =40)

**Figure 2:**
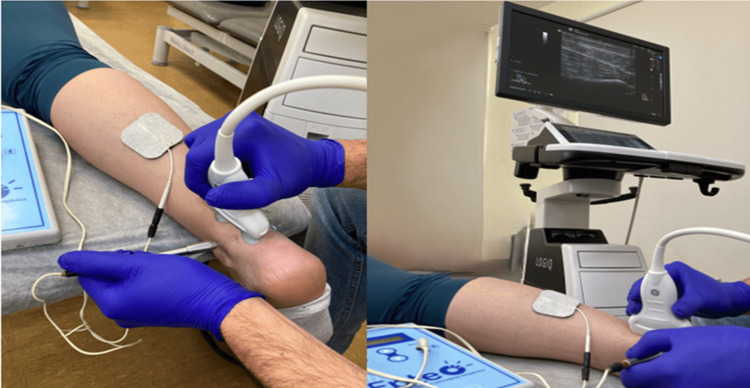
Ultrasound-guided percutaneous electrolysis technique.

The patient is placed in a prone position with the feet outside the stretcher. Before the procedure begins, the physiotherapist uses sterile gloves and examines the area to be treated using a linear ultrasound probe, performing a two-dimensional study in B mode and Doppler mode to identify the target tissue safely and ensure the effectiveness of the technique. The image is frozen to measure the distance from the skin to the target tissue to select the needle, which measures 0.30 × 25 mm in length (Agupunt, Madrid, Spain). The physiotherapist subsequently cleans the area thoroughly with sterile gauze and 2% chlorhexidine solution. The ultrasound probe is placed back over the target tissue, the needle is inserted into the handle, and the contact electrode is placed in the upper area near the needle to close the circuit. Once located in the area to be treated, the CE-certified PE apparatus is turned on for the percutaneous application of galvanic current EPTE® (IONCLINICS & DEIONIC SL, Av. Antonio Almela 29, 46250 L'Alcudia, Valencia, Spain) at 350 μA, and a treatment time of one minute and 20 seconds is applied by pressing the "ON" button to start the technique. The current rises progressively up to 350 μA once the intensity begins to decrease during the one minute and 20 seconds. At the end of the technique, the physiotherapist first removes the ultrasound probe and then removes the needle and compresses the area with sterile gauze for 30 seconds to prevent excessive bleeding. This intervention is shown in Figure 2.

This intervention was performed once a week for four weeks. The intervention exercises are described in Table 1.

**Table 1:** Common part of the treatment.

Intervention component	Description
Time of treatment	4 weeks (1 session per week)
Stretches	Stretch standing gastrocnemius (3 x 20 seconds); gastrocnemius seated stretch (3 x 20 seconds)
Therapeutic exercises eccentric	Calcaneal lowering knee slightly bent (2 x 15 reps 5’ lowered); calcaneal descent straight knee (2 x 15 reps 5’ lowered)

Statistical analysis was carried out using IBM SPSS Statistics for Windows, Version 28 (Released 2021; IBM Corp., Armonk, New York). A significance level of 0.05 was used for all tests. Initial comparisons between groups for qualitative variables were performed using Pearson’s chi-square test. For quantitative variables, initial comparisons between groups were performed using Student’s t-test for independent samples; the assumptions of normality (Shapiro-Wilk test) and homogeneity of variance (Levene test) were prechecked.

To determine the effectiveness of the treatments, two-factor ANOVA tests with repeated measures were performed using the linear model procedure. To study the effects on the dependent variables (scores on the scales), intrasubject factors (time: pre- and weekly measurements) and intersubject factors (treatment group) were considered, in addition to the interaction between both factors.

## Results

**Figure 3:**
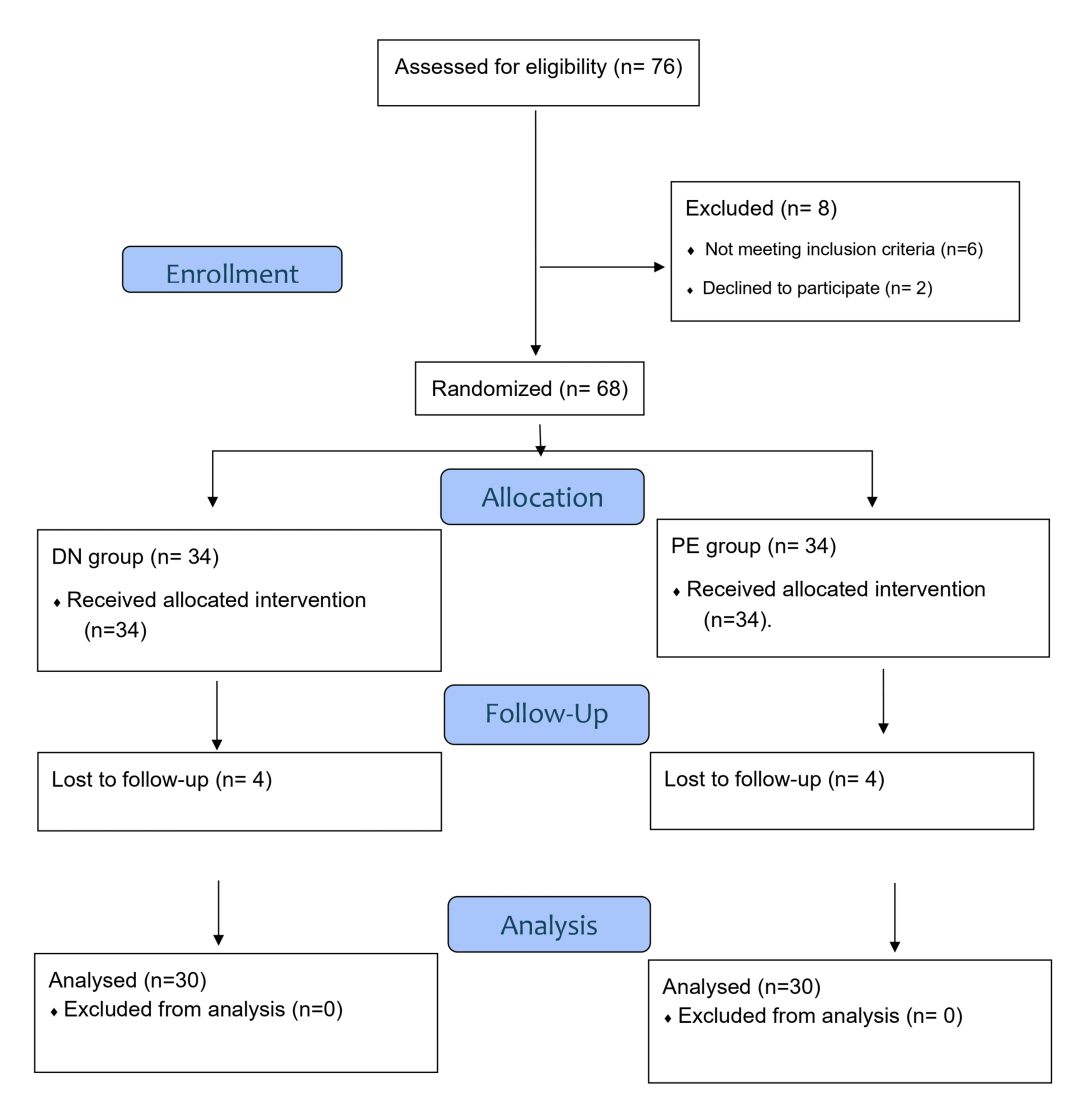
Flow diagram.

A total of 76 people were initially recruited for this research. Of these, six did not meet the inclusion criteria, one did not show up for the initial assessment, and one was excluded for other reasons; therefore, 68 people were ultimately randomly allocated, 34 people (n = 34) to the DN group and 34 people (n = 34) to the PE group. However, for different reasons, there were losses in both groups. In the DN group, there were four losses: not showing up for the last evaluation (n = 1), not tolerating the technique (n = 2), and absence without justification (n = 1). In the PE group, there were also four losses: not receiving the assigned intervention due to absence without justification at the first session (n = 1), not tolerating the technique (n = 1), and not showing up at the last assessment (n = 2). In the end, 60 people were evaluated and completed the intervention (Figure 3).

Table 2 shows the descriptive and comparative demographic and clinical variables between the groups.

**Table 2:** Descriptive and comparative demographic and clinical variables between the groups. PE: percutaneous electrolysis, DN: dry needling, X^2^: chi-square test.

Variables	Group		Test	p-value
	PE	DN		
Gender, n (%)			χ^2^(1) = 0.08	0.781
Men	21 (70)	20 (66.7)		
Women	9 (30)	10 (33.3)		
Age, mean (DT)	44.1 (9.8)	41.3 (11.5)	t(58) = 1.03	0.307
BMI, mean (DT)	24.6 (1.32)	25.48 (2.88)	t(58) = -1.52	0.134
Side, n (%)			χ^2^(1) = 0.60	0.438
Right	17 (56.7)	14 (46.7)		
Left	13 (43.3)	16 (53.3)		

**Figure 4:**
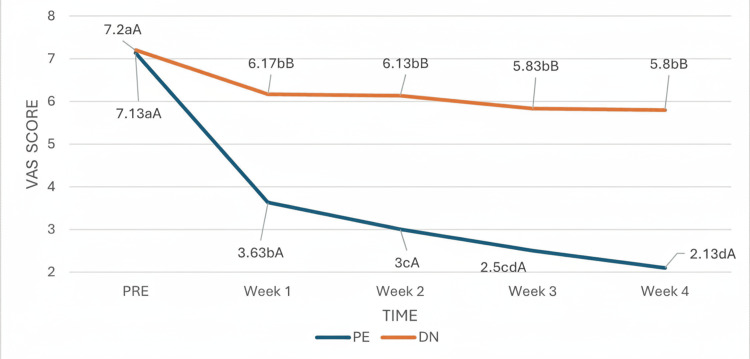
Group and time interaction effects on the VAS score. (a-d) Different lowercase letters indicate statistically significant differences between time points in the same group (Bonferroni correction). (A-B) Different capital letters indicate statistically significant differences between groups at the same time point (Bonferroni correction). VAS: Visual Analogue Scale.

With respect to the main study variable, the VAS score, the test revealed that the time effect was statistically significant, indicating that the subjective perception of pain as assessed by the VAS changed significantly over the course of the study (Figure 4). From week one onward and for the remaining weeks, in the comparison between the two intervention groups, the VAS score of the patients in the PE group was significantly lower than that of the patients in the DN group. However, there were no significant differences within either group when analyzed week by week; that is, the main difference occurred in the first treatment session, and this difference was maintained until the end of the study.

**Figure 5:**
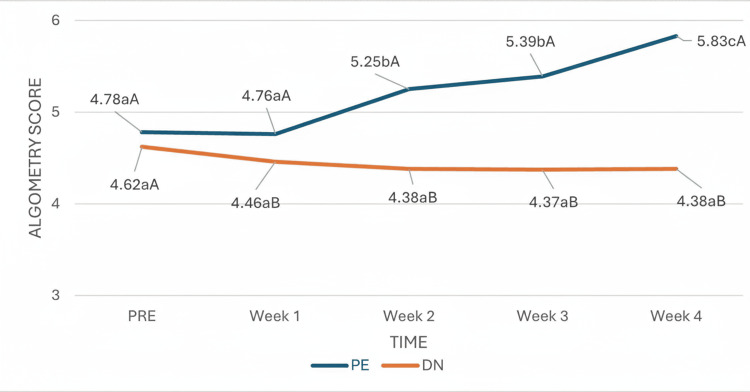
Interaction of group and time in Achilles tendinopathy algometry. (a-c) Different lowercase letters indicate statistically significant differences between time points in the same group (Bonferroni correction). (A-B) Different capital letters indicate statistically significant differences between groups at the same time point (Bonferroni correction).

In the AT algometry test, the time effect was statistically significant, indicating that the pressure pain threshold assessed by algometry changed significantly over the course of the study, regardless of group. However, there was a significant interaction effect between group and time, indicating that the passage of time influenced patients differently depending on the treatment group (Figure 5). From week two onward, when a significant change in the evolution of AT was observed in the PE group, and during the remaining weeks, the trend of evolution in both groups was clearly different, with the values of patients in the PE group being significantly greater than those of patients in the DN group.

**Figure 6:**
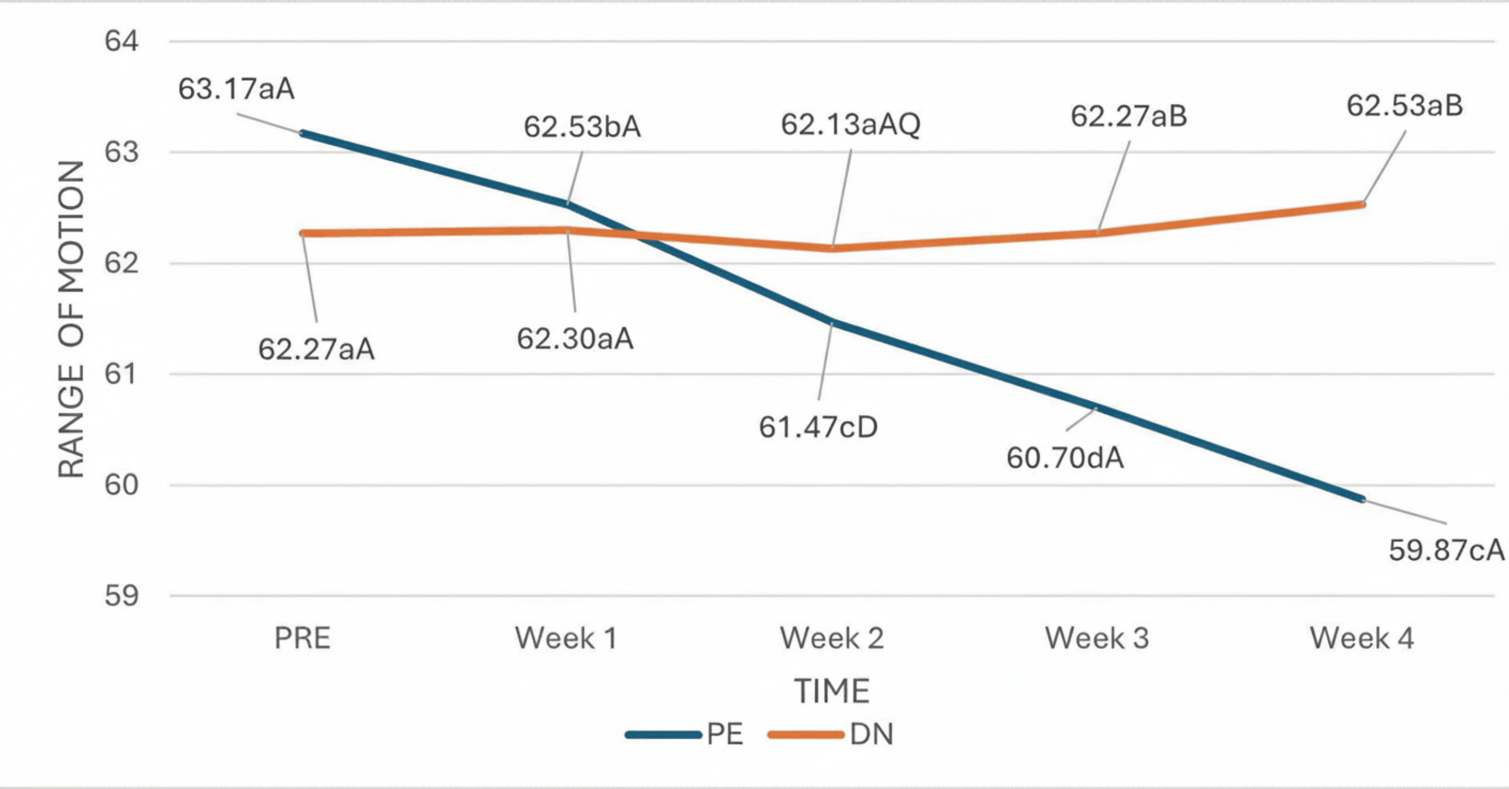
Interaction of group and time in goniometry. (a-c) Different lowercase letters indicate statistically significant differences between time points in the same group (Bonferroni correction). (A-B) Different capital letters indicate statistically significant differences between groups at the same time point (Bonferroni correction).

For ankle dorsiflexion goniometry, the test revealed that the time effect was statistically significant, and as with the previous variables, this finding indicates that the osteoarticular range of motion of ankle dorsiflexion assessed by goniometry changed significantly throughout the study (Figure 6), irrespective of group. However, there was a significant interaction effect between group and time, which indicates that the passage of time influenced patients differently depending on the treatment group, since the DN group remained constant without significant changes. From week two onward, when a significant change in the evolution of goniometry was observed in the PE group, and during the remaining weeks, the trend of evolution in both groups was clearly different, with the values of patients in the PE group being significantly greater than those of patients in the DN group.

**Figure 7:**
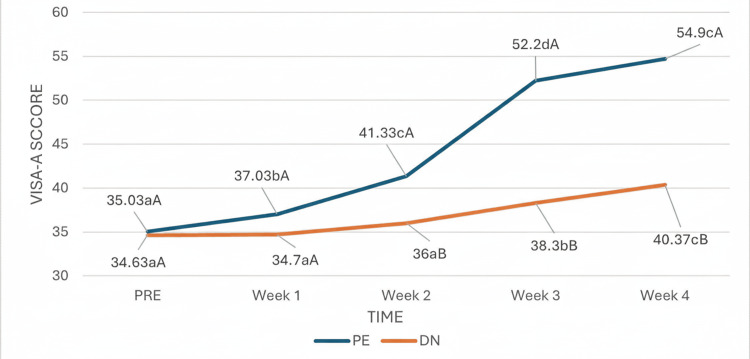
Interaction of group and time in the evaluation of the VISA-A. (a-c) Different lowercase letters indicate statistically significant differences between time points in the same group (Bonferroni correction). (A-B) Different capital letters indicate statistically significant differences between groups at the same time point (Bonferroni correction). VISA-A: Victorian Institute of Sport Assessment-Achilles.

With respect to the VISA-A scale, the time effect was statistically significant, indicating that quality of life in relation to pain in the Achilles tendon region changed significantly throughout the study, regardless of group. However, there was a significant interaction effect between group and time, indicating that the passage of time influenced patients differently depending on the treatment group (Figure 7). From week two onward, when a significant change in the evolution of VISA-A was observed in the PE group, and during the remaining weeks, the trend in the evolution of the groups clearly differed, with the values of patients in the PE group being significantly greater than those of patients in the DN group.

**Figure 8:**
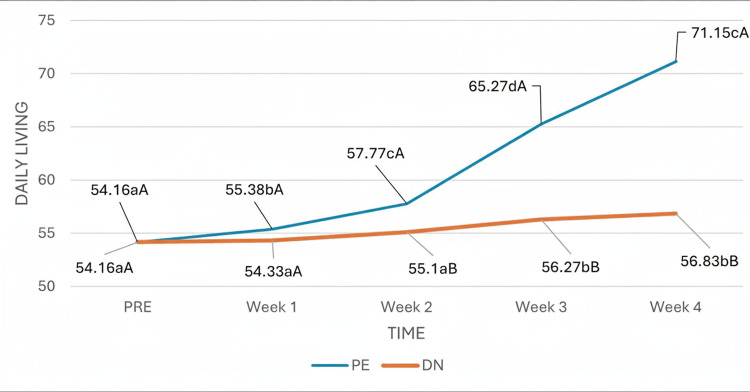
Interaction of group and time on the daily living subscale. (a-c) Different lowercase letters indicate statistically significant differences between time points in the same group (Bonferroni correction). (A-B) Different capital letters indicate statistically significant differences between groups at the same time point (Bonferroni correction).

In the daily living (DL) dimension of the FAAM scale, the test revealed that the time effect was statistically significant, indicating that the capacity and functionality of the ankle and foot, as measured by the FAAM DL subscale, changed significantly over the course of the study, regardless of group. However, there was a significant interaction effect between group and time, indicating that the passage of time influenced patients differently depending on the treatment group (Figure 8). From week two onward and for the remaining weeks, in the comparison between the two intervention groups, the DL subscale values of patients in the PE group were significantly greater than those of patients in the DN group.

**Figure 9:**
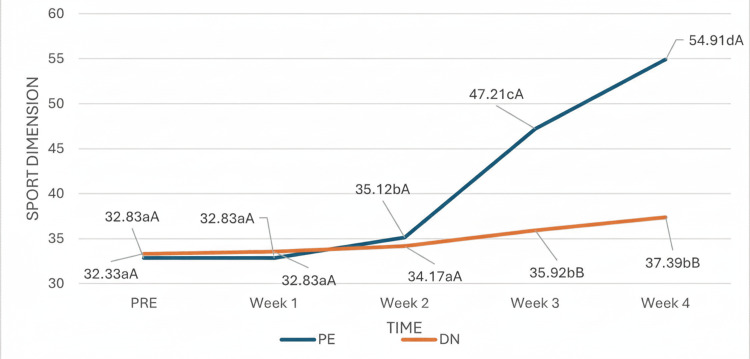
Group and time interaction in the sports subscale. (a-c) Different lowercase letters indicate statistically significant differences between time points in the same group (Bonferroni correction). (A-B) Different capital letters indicate statistically significant differences between groups at the same time (Bonferroni correction).

In the sports dimension of the FAAM scale, the time effect was statistically significant, indicating that the capacity and functionality of the ankle and foot, as measured by FAAM scale subscale D, changed significantly throughout the study (Figure 9), regardless of group. From week two, the week in which a significant change in the evolution of subscale D was observed in the PE group, and during the remaining weeks, the evolution trend in both groups was clearly different, where the values of the patients in the PE group were significantly greater than those of the patients in the DN group.

## Discussion

The main objective of this study was to analyze and compare the effects of applying DN to the MTrPs of the gastrocnemius muscle in the short and medium term on pain intensity compared with the effects of applying ultrasound-guided PE to the Achilles tendon in patients with AT.

A previous clinical trial compared these two techniques for the treatment of plantar pain in the heel and evaluated only pain, foot health, and quality of life [18]. Additionally, two other studies compared both techniques for the treatment of temporomandibular myofascial pain [19] and for pain in active myofascial trigger points in the levator scapulae muscle [20]. Fakontis et al. [13] reported that PE is more effective than DN in reducing pain, which is in line with the results presented in our study, but their results were not clinically significant. However, to our knowledge, this clinical trial is the first to compare the effectiveness of these techniques in terms of pain intensity, pain threshold to pressure, range of motion, quality of life, and functionality of the ankle and foot in patients with AT.

Several studies support the use of DN for the treatment of tendinopathies, as it has been shown to promote tendon recovery by increasing blood flow through local vasodilation and collagen proliferation [12]. There is strong evidence that DN is effective for the treatment of MTrPs. Hsieh and Baldry et al. [21] reported that insertion of a needle into the terminal plate reaching the local spasm response (REL) produces an increase in discharges, decreases acetylcholine reserves, and stimulates A-delta nerve fibers. This activates the inhibitory interneurons of the dorsal horn, lowering the levels of algogenic substances such as bradykinin and substance P [21]. Studies have shown that DN reduces pain intensity in patients with MTrPs during the week of treatment, and values during the month of treatment are maintained [18-20]. Our findings revealed that there was a significant effect of group and time interaction, which indicates that the passage of time influenced patients differently depending on the treatment group. In PE patients, pain intensity decreased significantly over time. Starting at week one and for the remaining weeks, the VAS score of patients in the PE group was significantly lower than that of patients in the DN group.

Our results revealed that the pressure pain threshold (PPT) in both the Achilles tendon and the gastrocnemius muscle MTrPs decreased until week one and week two and increased during the remaining weeks, but the difference was not statistically significant throughout the study. Studies have shown the importance of achieving the highest number of RELs, as this has been associated with a better clinical response. However, according to our study, obtaining REL during the technique may be clinically important but is not always synonymous with success [22,23]. Previously, other studies in which both techniques were compared have suggested that PE may be more effective than DN in the management of musculoskeletal pain [4,13,19,20]. In the present study, PPT values in both the Achilles tendon and gastrocnemius were significantly greater in the PE group than in the DN group, increasing more significantly from week two. These results coincide with the data revealed by Valera-Calero et al. [24], who reported statistically significant improvements with respect to PPT at seven days in the PE group, without finding statistically significant differences in the DN group.

One of the risk factors for AT is a movement deficit in ankle dorsiflexion and the subtalar joint. This deficit in range of motion causes a decrease in load tolerance in the tendon and generates abnormal movement patterns during loading by decreasing the mechanical properties of the tendon, thus contributing to the development of injury [25]. DN has been shown to increase lateral flexion range of motion and neck rotation in patients with neck pain and MTrPs in the levator scapulae muscle [20], and in patients with posterior shoulder stiffness, range of motion improved after the session [21], and in patients with temporomandibular disorders, mouth opening improved immediately after and during the week of treatment [19]. In contrast, our results coincide with those of Lake et al. [26], where no statistically significant differences were found regarding ankle dorsiflexion range of motion in patients who received DN over the triceps surae muscle. Within the limitations reflected in their study, healthy subjects were included who did not present a previous significant restriction in ankle dorsiflexion; therefore, they could not assess the influence that DN may have had on patients with limited movement. Another limitation was that they received only a single DN session and were evaluated after three days, and studies with longer follow-up are necessary to determine the benefits of DN and more consecutive sessions. Strengthening our results, we conducted a study in patients with heel pain due to plantar fasciitis and reported no statistically significant differences in ankle dorsiflexion range of motion after four weeks of treatment with DN; this study was limited by the small sample size, as only 20 participants were included [27]. In our study, despite having a larger sample size of 60 participants, we agree with previous studies that more long-term research is needed on the use of this technique to determine its effectiveness. Our results regarding ankle dorsiflexion ROM revealed that there was a significant effect of group and time interaction, which indicated that the passage of time influenced patients differently depending on the treatment group to which they belonged. In PE patients, ankle dorsiflexion ROM increased significantly from week two, whereas in the DN group, ROM remained unchanged throughout the study.

AT is accompanied by pain and impaired function in the Achilles tendon; therefore, it is considered necessary to assess the severity of AT in three fundamental aspects: pain, functional status, and activity. For this purpose, we used the VISA-A questionnaire and the FAAM scale.

Our results revealed that in both the VISA-A and the FAAM scales, the time effect was statistically significant, which indicates that both scales changed significantly throughout the study, independent of the group. However, there was a significant effect of group and time interaction, which indicates that the passage of time influenced patients differently depending on the treatment group. No study has compared this variable in this pathology; however, in contrast to our results, Benito et al. [28] compared both techniques in active MTrPs of the scapula lift, performing only one treatment session with a follow-up period of 14 days and reported statistically significant differences in favor of DN immediately after treatment. Nevertheless, at 72 hours and 14 days, there were no statistically significant differences between the two groups, possibly because only one intervention was carried out in their investigation and it was evaluated only in the short term [29]. On the other hand, with respect to function, in line with our results, López et al. [19] compared both techniques for the treatment of temporomandibular myofascial pain by three treatment sessions, each lasting one week, and reported that both techniques improved function and quality of life, but scores were higher and better in the PE group throughout the study.

The limitations of this study are several. The sample may not be sufficiently homogeneous, since the participants came from different settings, including sports clubs and private clinics. Second, the intervention lasted four weeks, and the results were measured in the short and medium term, so a longer-term evaluation would have been useful; at least three months would be needed to compare the effects and determine whether they would be sustained over time. Third, the intervention of this clinical trial was carried out by the same physiotherapist in both groups, which may limit the generalizability of the results. In addition, another important limitation with respect to the intervention is that the DN technique was performed on the most hyperalgesic MTrPs of the gastrocnemius muscle, whereas the PE technique was performed on the most painful point of the Achilles tendon. Therefore, for future research, it would be interesting to compare both techniques performed in the same area, even combining them and adding more patients to the study, with a more homogeneous sample.

## Conclusions

Echoguided PE applied to the Achilles tendon is more effective than dry puncture applied to the MTrPs of the gastrocnemius muscle in reducing pain intensity and short- and medium-term mechanosensitivity in patients with AT.

In addition, echoguided PE applied to the Achilles tendon is more effective than DN applied to the MTrPs of the gastrocnemius muscle in increasing ankle dorsiflexion ROM and improving function and quality of life in the short and medium term in patients with AT.
